# Minimizing Surgical Margins in Basal Cell Carcinoma: A Single Institution's Experience with Excision and Reconstruction Methods

**DOI:** 10.1055/s-0044-1788780

**Published:** 2024-12-27

**Authors:** Sang-Oh Lee, Tae Gon Kim, Kyu Jin Chung

**Affiliations:** 1 Department of Plastic and Reconstructive Surgery, Yeungnam University College of Medicine, Daegu, Republic of Korea

**Keywords:** basal cell carcinoma, wide excision, frozen biopsy, reconstruction

## Abstract

**Background** Basal cell carcinoma (BCC) is the predominant nonmelanocytic skin cancer, with preservation of both function and aesthetics being essential during tumor removal. Existing surgical margin guidelines primarily target ill-defined BCCs prevalent in Western countries. Therefore, this study aims to demonstrate the efficacy of surgical removal, propose modified guidelines for wide excision tailored to Asian patients, and share experiences with various reconstruction methods.

**Methods** This study encompasses 418 patients (447 cases) who underwent BCC excision from March 2015 to June 2023 at our institution. Wide excision extended 2 mm beyond the tumor edge universally, with an additional 2 mm resected if tumor cells persisted in the frozen biopsy, followed by appropriate reconstruction. Patient demographics, tumor features, reconstruction methods, complications, and recurrence rates were analyzed.

**Results** Predominantly, reconstructions involved local flaps (244), skin grafts (102), and direct closure (72). Significant differences were noted in age, location, and tumor size among these groups. The rate of second resection increased from upper to lower facial subunits, peaking at 11.1% in the lower subunit, with a statistically significant difference ( *p* = 0.024). Additional resection was required in 5.50% of cases, with a significantly higher incidence of ill-defined borders, pigmentation, and the infiltrative subtype compared with others. Complications were minor; recurrence occurred in only one case, 6 months postinitial nasal dorsum surgery.

**Conclusion** Surgical excision is highly effective, supported by various reconstruction options. We propose narrower guidelines for wide excision considering tumor characteristics and recurrence locations, resulting in smaller defects addressed with simpler reconstruction methods.

## Introduction

Basal cell carcinoma (BCC) is recognized as the most prevalent type of nonmelanocytic skin cancer. It originates from the basal layer of the epidermis and is characterized by its locally invasive and slowly spreading nature. 1 2 Notably, BCC exhibits an extremely low tendency for metastasis, with the incidence of metastasis estimated to be between 0.0028 and 0.055%. 3 Annually, there are approximately 3.6 million cases diagnosed worldwide. In the United States, around 2,000 deaths per year are attributed to BCC. 4

BCC can be suspected based on certain visible characteristics. These include red patches accompanied by itching, a scar-like appearance, an ulcer that fails to heal or recurrently appears, and pink or red bumps that might have blue, brown, or black areas. Such clinical presentations are key indicators for considering a diagnosis of BCC. 5 Additionally, BCC can be categorized into various histopathologic types, including nodular, superficial, infundibulocystic, fibroepithelial, morpheaform, infiltrative, micronodular, and basosquamous types. 6 It is noteworthy that some metastatic BCC cases exhibit features of squamous differentiation or present a poorly differentiated pattern. 7

Surgical excision is considered the mainstay of treatment for BCC, with a reported 5-year disease-free rate exceeding 98%. 8 According to the National Comprehensive Cancer Network (NCCN) guidelines (Version 2.2024) for surgical removal of BCC, the standard excision with 4 mm clinical margins is recommended for low-risk BCC, while an excision with clinical margins wider than 4 mm is advised for high-risk BCC.

Mohs surgery is preferred for its ability to spare normal tissue as much as possible, offering favorable functional and cosmetic outcomes. 9 However, the practical implementation of this technique presents challenges. The lengthy and technically demanding training process required for surgeons entails significant time investment. Furthermore, the need for specialized facilities to conduct pathologic evaluations during the surgery adds to the complexity. As a result, it is not always feasible to perform the surgery in many clinical settings. Furthermore, in situations where frozen biopsy is available, there is a lack of established guidelines on how to determine the resection margins.

Considering the lower metastasis rates and less invasive nature of BCC, effective treatment is highly achievable when the tumor is detected and diagnosed early. The primary goal of this study is to demonstrate that, in settings where frozen biopsy is available, performing wide excision with even smaller clinical margins than current guidelines recommend can still result in low recurrence rates. This approach also simplifies the reconstruction methods needed to address the defects created by excision, thereby showcasing the effectiveness of surgical removal. Additionally, the secondary goal is to share the experience of a single institution with various reconstruction methods, offering guidance in selecting appropriate reconstructive techniques.

## Methods

From March 2015 to June 2023, a retrospective study was conducted at our hospital, involving 422 patients who underwent wide excision for BCC. Informed consent was obtained from all participants. The surgeries were performed by a single surgeon (K.J.C.). The inclusion criteria for this study were patients undergoing their first surgery for the specified BCC and those without suspected regional lymph node metastasis on physical examination. Patients excluded from the study were those who had received prior treatments such as radiotherapy, cryotherapy, immunotherapy, or other topical treatments for BCC, as well as immunocompromised patients or those on immunosuppressive medications. Consequently, the study encompassed 447 cases involving 418 patients.

Data collected included the patient's age, gender, place of residence, methods of reconstruction performed following the wide excision, the necessity of additional frozen biopsies, tumor size (greatest dimension) and location, stage, underlying diseases, body mass index (BMI), recurrence status, follow-up duration, smoking history, and history of preoperative radiotherapy (not for BCC).

In patients who underwent surgery for BCC occurring on the face, the study investigated whether there were differences in the necessity of additional frozen biopsies based on the subunit of the face where the tumor occurred, as well as factors such as age, size, and BMI. Additionally, the study analyzed variations in surgical methods depending on the facial subunit affected by the tumor, as well as differences based on gender, age, BMI, and tumor size. The facial subunits were categorized into upper, middle, and lower units, delineated by a horizontal line extending from the subbrow margin and a curve formed by the subnasale, nasolabial fold, upper margin of mandible, and inferior margin of zygoma. The upper unit included the frontal and parietal regions; the middle unit included the temporal region, periorbital region, nose, zygoma, cheek, lower cheek, and ear, while the lower unit included the perioral region and chin. 10 This study was approved by the Institutional Review Board of Yeungnam University Hospital (IRB Number YUMC 2024-01-002).

### Surgical Procedures

After visually identifying the boundaries of the BCC, resection was conducted with a 2-mm margin from the edge of the boundary, irrespective of the varying risk group criteria outlined in the NCCN guidelines. The depth of resection aimed to be as minimal as possible, targeting the suprafascial plane. To determine the necessity of additional resection, the excised tumor was tagged at the 3, 6, 9, and 12 o'clock positions and the base, followed by a frozen biopsy. If the frozen biopsy revealed a residual tumor, further resection was performed in the remaining direction with a 2-mm margin, continuing until no residual tumor was detected in the frozen biopsy. The resected specimens were fixed in formalin and sent to pathology for margin evaluation, involving processing and staining. Upon confirmation of no residual tumor in the frozen biopsy after the wide excision, the defect's location and size were considered, and appropriate reconstruction methods such as direct closure, local flap, or skin graft were employed to cover the defect. Additionally, the surgical process, including preoperative, intraoperative, and postoperative stages, was documented photographically. Recurrence was defined as the pathological confirmation of BCC in cases where lesions suspected to be BCC, such as scars or ulcer-like lesions, nonhealing wounds, or nodules with redness, blue or black pigmentation, appeared at the site of the previous surgery.

### Statistical Analysis

Data analysis was conducted using IBM SPSS Statistics for Windows, version 22.0 (IBM Corp., Armonk, NY). In the analysis of variations in the necessity of additional frozen biopsies among BCC patients based on various factors, the Mann–Whitney test and Fisher's exact test were utilized. For examining differences in the reconstruction method after the wide excision according to different variables, statistical analysis was performed using the ANOVA test, followed by post hoc tests. In all statistical analyses, a *p* -value of less than 0.05 was considered statistically significant.

## Results

Our institution reviewed the medical records of 447 cases involving 418 patients. The average age was 74.62 years (range, 37–102), with females accounting for 52.4% of the total cases. The average size of the tumor (greatest dimension) was 0.96 cm (range, 0.1–4.4), the average BMI was 23.48 kg/m ^2^ (range, 5.43–37.10), and the average follow-up period was 7.57 months (range, 0.25–62). Skin type III was the most prevalent (93.51%), and tumors were more commonly well-defined (85.23%) and pigmented (65.55%; Table 1 ).

**Table 1:** Patient demographics

Variable	*N* (range or percent)
Age, years	74.62 (37–102)
Female, *n*	234 (52.40)
BMI, kg/m ^2^	23.48 (15.43–37.10)
Ethnicity, *n*	
Asian	444 (99.33)
White	3 (0.67)
Skin type, *n* a	
Type I	3 (0.67)
Type III	418 (93.51)
Type IV	26 (5.82)
Tumor size, cm	0.96 (0.1–4.4)
Stage (T)	
I	414 (92.62)
II	33 (7.38)
Border	
Well-defined	381 (85.23)
Ill-defined	66 (14.77)
Pigmented	293 (65.55)
Follow-up period, months	7.57 (0.25–62)
Radiation therapy (preoperative, not for BCC)	2 (0.48)

Abbreviations: BCC, basal cell carcinoma; BMI, body mass index.

a Fitzpatrick skin type.

The location of BCC occurrence predominantly involved the face, with 382 cases noted in this region. Within the facial cases, the nose was the most common site, accounting for 144 instances, with the highest frequency observed on the nasal dorsum, followed by the alar and tip. After the nose, the cheek and auricle were the next most frequent sites. The occurrences of BCC on other body regions were comparatively fewer, with 6 cases on the neck, 17 on the trunk, and 6 cases each on the upper and lower extremities ( Supplementary Table S1 [available in the online version only]).

Twenty-three cases, accounting for 5.15% of the total, required additional frozen biopsies during wide excision due to the presence of residual tumors. There were no instances where frozen biopsies were performed more than twice. When looking at the rate of second resection based on facial subunits, it becomes evident that the rate of second resection increased as we moved from upper to lower subunits with the highest rate observed in the lower subunit (11.1%). The difference in the number of additional resections performed across the facial subunits was statistically significant ( *p* = 0.024; Table 2 ). When comparing patient and tumor characteristics between cases requiring additional resection and those that did not, statistically significant differences were observed in tumor border, pigmentation, and histological subtype, except for skin type. Cases requiring additional resection had a higher proportion of ill-defined borders and pigmentation (43.5 vs. 14.9%; *p* = 0.002, 100 vs. 63.68%; *p* = 0.000). Furthermore, the proportion of the infiltrative subtype was significantly higher compared with other subtypes in the cases (43.5%; *p* = 0.004; Table 3 ). The preoperative photos of patients who underwent additional excision are presented in Supplementary Fig. S1 (available in the online version only).

**Table 2:** Second resection rates according to facial subunits

	No second resection ( *N* [%])	Second resection ( *N* [%])	*p* -Value
Subunit			0.024 a
Upper	63 (100)	0 (0)	
Middle	272 (93.2)	20 (6.8)	
Lower	24 (88.9)	3 (11.1)	
Total	359 (94)	23 (6)	

a *p* < 0.05.

**Table 3:** Characteristics differences between groups with and without additional resection

	No second resection, *n* (%)	Second resection, *n* (%)	*p* -Value
Skin type			0.475
Type I	3 (0.7)	0 (0)	
Type III	395 (93.2)	23 (100)	
Type IV	26 (6.1)	0 (0)	
Tumor border			0.002 a
Well-defined	361 (85.1)	13 (56.5)	
Ill-defined	63 (14.9)	10 (43.5)	
Pigmented	270 (63.68)	23 (100)	0.000 a
Histopathological subtypes			0.004 a
Nodular	212 (50.0)	6 (26.1)	
Micronodular	54 (12.7)	1 (4.3)	
Superficial	69 (16.3)	4 (17.4)	
Infiltrative	85 (20.1)	10 (43.5)	
Morpheaform	4 (0.9)	2 (8.7)	

a *p* < 0.05.

**Fig. 1:**
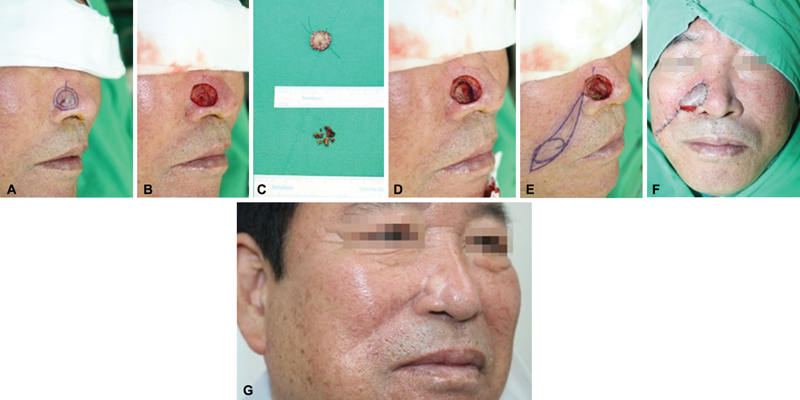
A case of interpolation flap following wide excision of BCC. ( **A, B** ) A 67-year-old male patient with a 15-mm BCC on the Rt. nasal alar underwent wide excision. ( **C–E** ) A residual tumor was detected at the base resection margin of the specimen. A 2-mm additional resection was performed in the direction of the base, which included the right upper lateral cartilage. ( **F** ) An interpolation flap was used, and 1 month later, flap division was performed. ( **G** ) There was no recurrence within the first 2 years postsurgery. BCC, basal cell carcinoma; Rt., right.

Once the absence of residual tumor was confirmed via frozen biopsy, various methods were employed for the reconstruction of defects following wide excision ( Fig. 1 ). The most common techniques were local advancement flap, skin graft, and direct closure, used in 119 cases (28.47%), 102 cases (24.40%), and 72 cases (17.22%), respectively. Among local flap methods, excluding the local advancement flap, the bilobed flap, transposition flap, and V–Y advancement flap were the most frequently used in 66 cases (15.79%), 23 cases (5.50%), and 22 cases (5.26%), respectively ( Supplementary Table S2 and Supplementary Figs. S2–S4 [available in the online version only; *which demonstrate the cases of a skin graft, a bilobed flap, and a transposition flap, respectively* ]). Complications arising from the use of local flaps and skin grafts were minor, and are detailed in Supplementary Table S3 (available in the online version only). Recurrence occurred in only one of the total cases, specifically in a case of BCC on the nasal dorsum.

When comparing the groups that underwent direct closure and skin graft, it was observed that the age and size were significantly larger in the skin graft group. When considering facial subunits, the upper subunit showed the highest rate of skin graft (34.92%) compared with the other two subunits, while the lower subunit had the lowest rate of skin graft (7.41%) and the highest rate of direct closure and local flap ( Supplementary Table S4 , available in the online version only).

## Discussion

The NCCN guidelines categorize the low-risk group as those tumors occurring on the trunk or extremities, under 2 cm in size, with well-defined borders, usually primary tumors, and without a history of immunosuppression or radiotherapy. This group encompasses subtypes such as nodular, superficial, and nonaggressive patterns including keratotic, infundibulocystic, and fibroepithelioma of Pinkus. The high-risk group includes tumors that occur on the head, neck, hands, feet, pretibial, anogenital areas, or the trunk and extremities when over 2 cm. These tumors feature poorly defined borders, are often recurrent, occur in patients with a history of immunosuppression or radiotherapy, and include aggressive subtypes like basosquamous, infiltrative, sclerosing/morpheaform, micronodular, and BCC with carcinosarcomatous differentiation. The treatment for advanced BCC with nodal or distant metastasis is not definite, but surgery and systemic therapy can be considered. 11

Mohs surgery is considered unique in its ability to ensure complete tumor removal while demonstrating a very low recurrence rate. In the treatment of primary BCC, Mohs microscopic surgery (MSS) has demonstrated a significantly lower 5-year recurrence rate of 1.0% compared with the 10.1% observed with standard excision. 12 Furthermore, in the case of recurrent BCC, the recurrence rate for traditional excision methods stands at 17.4%, whereas MMS shows a markedly reduced rate of 5.6%. 13 However, the extensive training required for surgeons and the specific facility requirements mean that MMS cannot be implemented in all hospitals. Consequently, in situations where the surgery is not feasible, there is no consensus on how to determine the extent of wide excision.

The aforementioned guidelines are primarily tailored for the Caucasian population, where lesions typically present as nonpigmented or with ill-defined borders. 14 In contrast, in Asians, 52.4 to 90% of BCC cases present as pigmented lesions, making the tumor margins more discernible. Consequently, various studies have investigated the efficacy of narrower surgical margins in this population. 15 A study conducted in Japan found that among 288 pigmented BCCs, the complete removal rates with 2 and 3 mm surgical margins were 95.3 and 100%, respectively. 16 Furthermore, a study involving 1,000 BCC cases in Japan included all types of BCCs, unlike previous studies. It found that when 2 and 3 mm surgical margins were used, the only factor that statistically significantly influenced the difference in estimated side margin positivity rates was the clarity of the tumor border, whether well- or ill-defined. This suggests that the clarity of the clinical tumor border is a reliable indicator for determining surgical margins. 15 Currently, a nonrandomized and prospective study is underway to compare the efficacy and safety of 2 and 3 mm surgical margins based on whether the tumor border is well- or ill-defined. Additionally, studies have explored the correlation between factors such as anatomical region, tumor thickness, histologic pattern, and tumor invasive level for setting deep surgical margins rather than width. 17 18 A recent systematic review reported that in low-risk patients with well-demarcated BCCs smaller than 2 cm, resection with a 3-mm clinical margin yielded satisfactory outcomes. In high-risk patients with BCCs larger than 2 cm, a margin of 4 to 6 mm showed favorable results. The review also suggested that, with close follow-up, it might be feasible to perform resections with a 2-mm clinical margin in low-risk groups, particularly for lesions that are clinically well-defined and where the traditional wide excision is impractical due to anatomical location or cosmetic considerations. 19

For Korean BCC patients, who are also of Asian descent, tumor margins are relatively easier to identify. However, there is limited information on how to set clinical surgical margins when performing traditional surgical excision, rather than MMS. In one study that conducted frozen biopsies, tumors were classified into low- and high-risk groups based on their gross appearance. In the low-risk group, resections were performed with a 2-mm clinical margin, resulting in a recurrence rate of 1.5%, while in the high-risk group, resections were performed with a 3-mm clinical margin, resulting in a recurrence rate of 2%. 20

In our study, the group that underwent additional excision showed a statistically significant higher prevalence of tumors with ill-defined borders, pigmentation, and the infiltrative subtype, known to be more invasive compared with other subtypes. 21 Additionally, the highest rate of additional excision was observed in the lower subunit of the face, with the sole recurrence case being a BCC on the nose. These findings suggest that for tumors exhibiting features such as ill-defined borders, pigmentation, or dermoscopic signs suggestive of infiltrative characteristics (arborizing vessels, ulceration, and short-fine telangiectasia 22 ), originating in the lower subunit of the face, or located on the nose, considering an initial wide excision with a clinical margin slightly larger than 2 mm might prevent the need for additional resections. This approach could lead to fewer tissue deficits.

When comparing the direct closure group with the skin graft group, the size of the defect was significantly larger in cases that required skin grafts, indicating that more invasive procedures like skin grafts are necessary as the size of the defect increases. Observing surgical method differences based on facial subunits, BCCs located in the lower subunit had the highest proportion of direct closure or local flap usage compared with the other two subunits. Conversely, BCCs in the upper subunit showed a higher tendency for skin grafting compared with the middle and lower subunits. This appears to be related to the mobility and laxity of the tissues in the middle and lower subunits, suggesting that defects in these areas can be addressed more simply.

However, there are several limitations to this study. The average follow-up period was 7.57 months, but there were cases with less than 6 months of follow-up. In such cases, it is difficult to assume no recurrence. Furthermore, small recurrences, especially those appearing as scar-like lesions, may be challenging to detect. Second, the inclusion criteria were set for cases without suspected metastasis, potentially leading to a selection bias toward lower-stage cases. Lastly, the statistical analysis of the main variables was conducted only for facial BCC cases. The number of cases occurring in other areas was too small for inclusion in the statistical analysis. Therefore, further research involving a larger collection of BCC cases occurring in other areas is necessary.

In a single-institution study involving a considerable number of patients with BCC affecting all body regions, performing wide excision with a 2-mm margin resulted in a low necessity for additional frozen biopsies and low recurrence rates. Based on these findings and other results presented in this study, preserving as much healthy tissue as possible can lead to less invasive reconstruction methods. Therefore, in institutions where Mohs surgery is not feasible but frozen biopsy is available, performing wide excision with a smaller 2-mm margin for BCC patients, as opposed to the larger margins recommended in existing guidelines, is a viable option. However, it may still be necessary to consider slightly increasing the margin beyond 2 mm depending on the border, pigmentation, and suspected subtype of the tumor. This approach can sufficiently achieve smaller postexcision defects and maintain low recurrence rates, offering an effective alternative to more extensive surgical methods.
