# Supplementary material for: Minimizing Surgical Margins in Basal Cell Carcinoma: A Single Institution's Experience with Excision and Reconstruction Methods
Source: Arch Plast Surg. 2024 Dec 27;52(1):30–5. doi: 10.1055/s-0044-1788780 (PMC11750340; doi:10.1055/s-0044-1788780)
Supplement: Supplementary file 1 — Supplementary Material [file 10-1055-s-0044-1788780-s24jan0014oa.pdf]

Supplementary Material

Supplementary Table S1 Locations of basal cell carcinoma

Locations	*N*
Face	382
Nose<br>Cheek<br>Auricle<br>Neck	Nasal dorsum<br>(64) > Alar (53) > Tip (21)<br>62<br>29<br>6
Trunk	17
Anterior chest<br>Back<br>Suprapubic area<br>Scrotum	8<br>6<br>2<br>1
Upper extremity	6
Axilla<br>Upper arm<br>Hand	2<br>3<br>1
Lower extremity	6
Buttock<br>Lower leg	3<br>3

Supplementary Table S2 Reconstruction methods

Methods	*N* (%)
Local advancement flap<br>Skin graft<br>Direct closure	119 (28.47)<br>102 (24.40)<br>72 (17.22)
Bilobed flap	66 (15.79)
Transposition flap<br>V–Y advancement flap<br>Yin and Yang flap<br>Interpolation flap<br>Composite graft<br>Paramedian forehead flap<br>Propeller flap<br>Rhomboid flap<br>Tenzel flap<br>Antia-Buch flap	23 (5.50)<br>22 (5.26)<br>3 (0.72)<br>2 (0.48)<br>1 (0.24)<br>1 (0.24)<br>1 (0.24)<br>1 (0.24)<br>1 (0.24)<br>1 (0.24)
Local advancement flap + Bilobed flap<br>Local advancement flap + Skin graft<br>V–Y advancement flap + Bilobed flap	1 (0.24)<br>1 (0.24)<br>1 (0.24)

Supplementary Table S3 Complications

Methods	*N* (%)
**Local flap**<br>Hypertrophic scar<br>Milium<br>Tissue necrosis<br>Seroma	244 (100)<br>7 (2.87)<br>2 (0.82)<br>2 (0.82)<br>1 (0.41)
Wound dehiscence<br>Bulky flap	1 (0.41)<br>1 (0.41)
**Skin graft**<br>Hyperpigmentation<br>Hematoma	102 (100)<br>11 (10.78)<br>1 (0.98)

 © 2024. The Author(s).

Supplementary Table S4 Factors associated with methods of reconstruction

Characteristic	Total ($n = 418$)	Direct closure ($n = 72$)	Local flap ($n = 244$)	Skin graft ($n = 102$)	p-Value
Sex					0.196
Male	199 (47.6%)	32 (44.4%)	125 (51.2%)	42 (41.2%)	
Female	219 (52.4%)	40 (55.6%)	119 (48.8%)	60 (58.8%)	
Age, years	74.6 ± 11.9	73.2 c 11.8[a]	73.8 ± 11.9[a,b]	77.6 ± 11.7[b]	0.014[c]
BMI, kg/m^2	23.4 ± 3.0	23.1 ± 2.9	23.7 ± 3.0	23.1 ± 3.0	0.301
Size, cm	0.96 ± 0.58	0.91 ± 0.79[a]	0.89 ± 0.48[a]	1.17 ± 0.57[b]	0.000[c]
**Characteristic**	**Total ($n = 382$)**	**Direct closure ($n = 60$)**	**Local flap ($n = 222$)**	**Skin graft ($n = 100$)**	**p-Value**
Location (face)					0.005[c]
Upper	63 (16.5%)	14 (23.3%)	27 (12.2%)	22 (22.0%)	
Middle	292 (76.4%)	38 (63.3%)	178 (80.2%)	76 (76.0%)	
Lower	27 (7.1%)	8 (13.4%)	17 (7.6%)	2 (2.0%)	

Abbreviation: BMI, body mass index.
[a,b]Post hoc.
[c]$p < 0.05$.

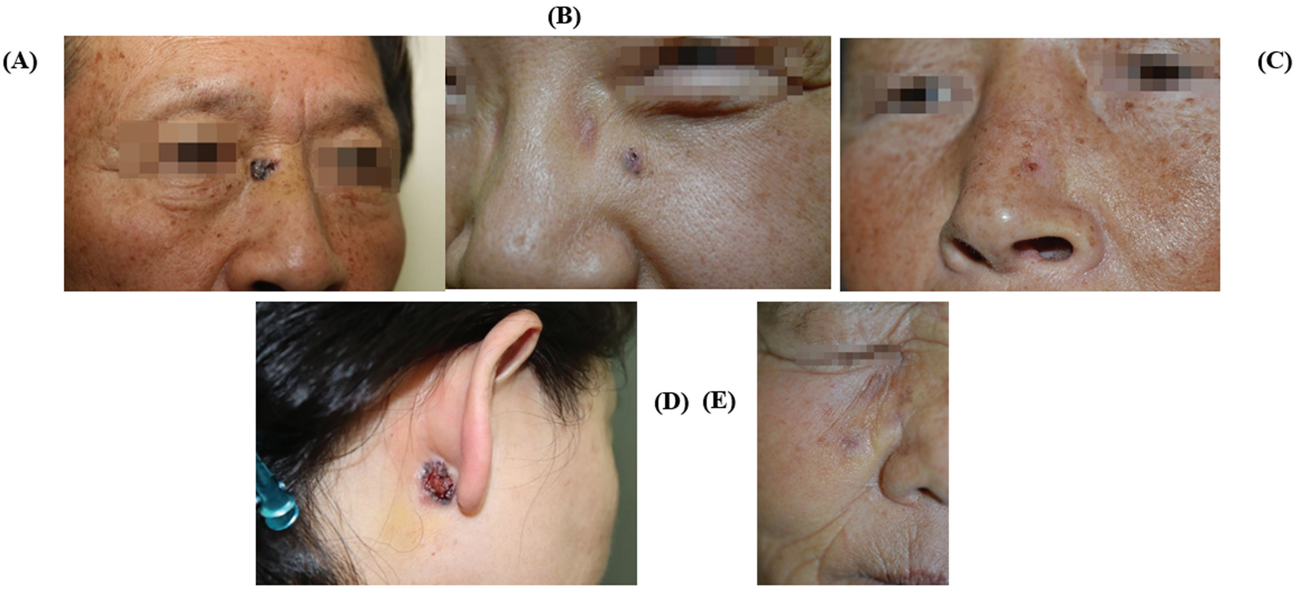

Supplementary Fig. S1 Tumor characteristics including border, pigmentation, and subtype. (**A**) Well-defined pigmented nodular BCC. (**B**) Well-defined pigmented micronodular BCC. (**C**) Ill-defined pigmented superficial BCC. (**D**) Well-defined pigmented infiltrative BCC. (**E**) Ill-defined pigmented morpheaform BCC. BCC, basal cell carcinoma.

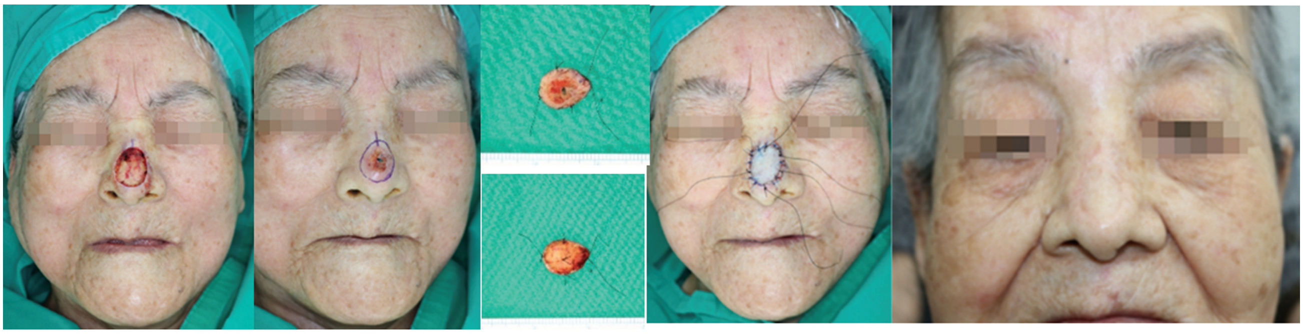

Supplementary Fig. S2 A 98-year-old female patient with a 15-mm BCC on the nasal dorsum underwent wide excision. With no tumor detected on frozen biopsy, a full-thickness skin graft was then performed using the right clavicle as the donor site. There was no recurrence within the 6 months following the surgery. BCC, basal cell carcinoma.

 © 2024. The Author(s).

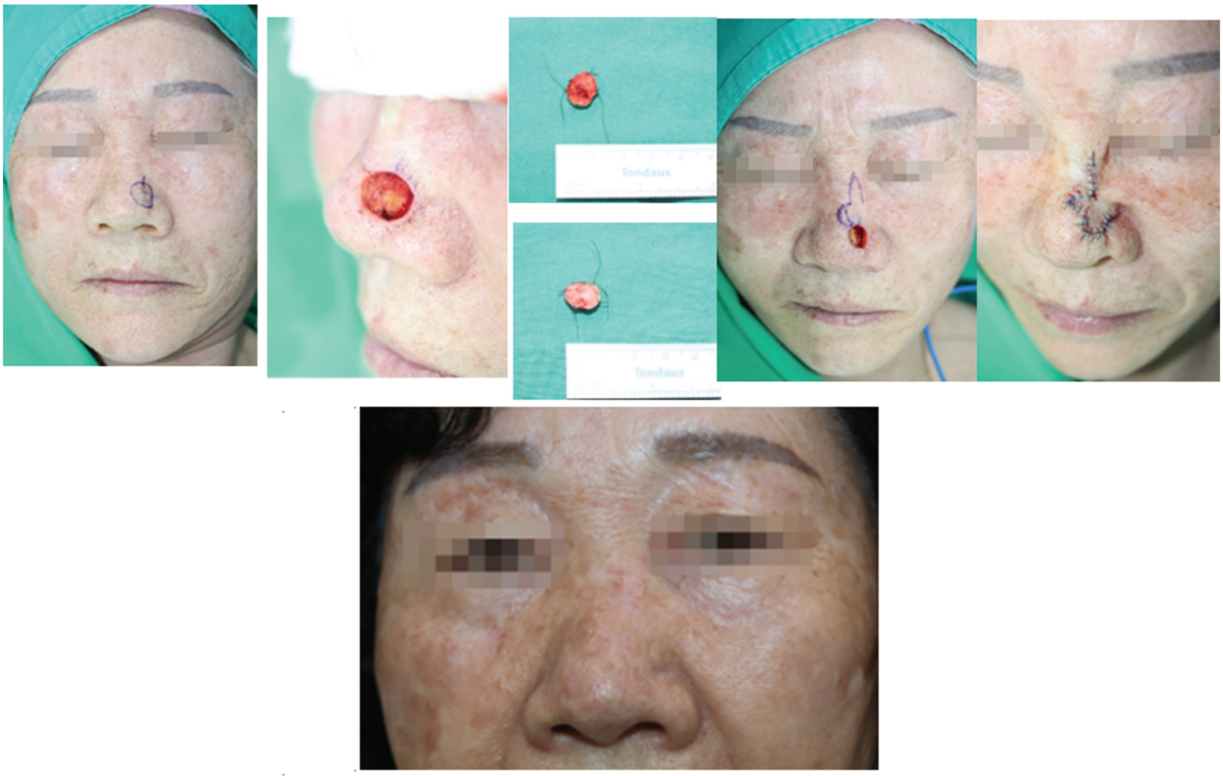

Supplementary Fig. S3 A 72-year-old female patient with a 10-mm BCC on the Lt. nasal alar underwent wide excision. With no remnant tumor detected in the frozen biopsy, a bilobed flap was performed, and there was no recurrence within 1-year postsurgery. BCC, basal cell carcinoma; Lt., left.

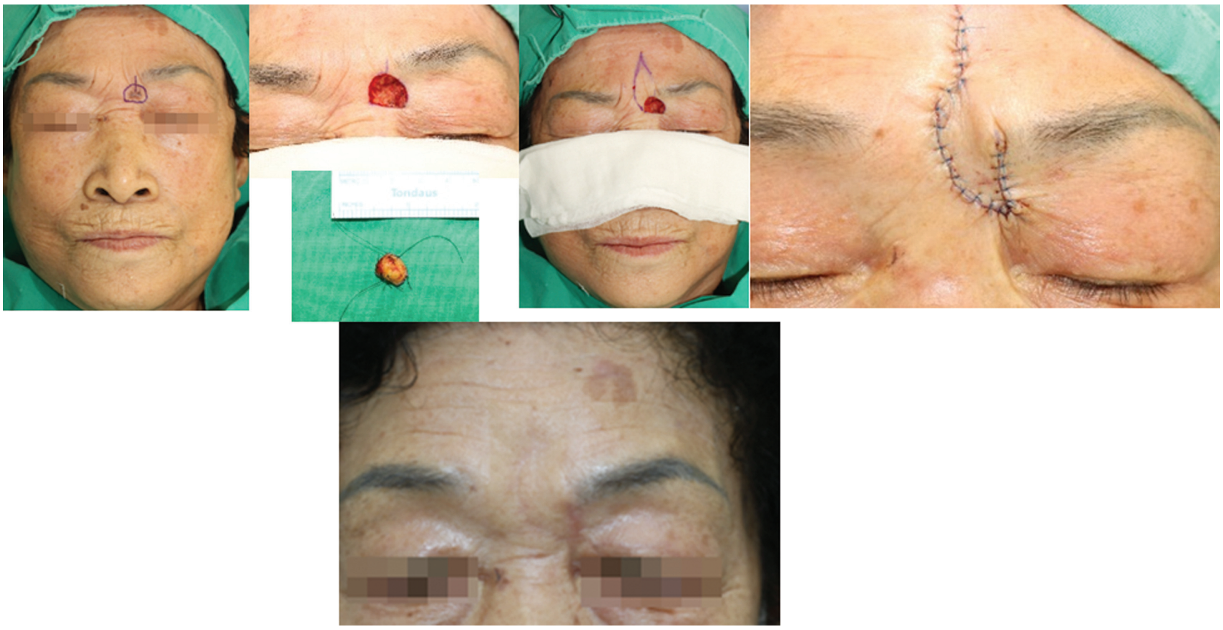

Supplementary Fig. S4 A 9-mm BCC on Lt. eyebrow, wide excision was performed and with no remnant tumor detected in the frozen biopsy, a transposition flap was performed. No recurrence occurred within the 6 months. BCC, basal cell carcinoma; Lt., left.

 © 2024. The Author(s).
